# Integration of Transcriptomic and Metabolomic Data to Compare the Hepatotoxicity of Neonatal and Adult Mice Exposed to Aristolochic Acid I

**DOI:** 10.3389/fgene.2022.840961

**Published:** 2022-03-25

**Authors:** Zhi-e Fang, Chunyu Wang, Ming Niu, Tingting Liu, Lutong Ren, Qiang Li, Zhiyong Li, Ziying Wei, Li Lin, Wenqing Mu, Yuan Gao, Xiaohe Xiao, Zhaofang Bai

**Affiliations:** ^1^ School of Pharmacy, Chengdu University of Traditional Chinese Medicine, Chengdu, China; ^2^ Department of Hepatology, Fifth Medical Center of Chinese PLA General Hospital, Beijing, China; ^3^ China Military Institute of Chinese Materia, Fifth Medical Center of Chinese PLA General Hospital, Beijing, China; ^4^ School of Traditional Chinese Medicine, Capital Medical University, Beijing, China

**Keywords:** aristolochic acid I, liver injury, neonatal, adult, metabolomics, transcriptomics

## Abstract

Aristolochic acid (AA) is a group of structurally related compounds what have been used to treat various diseases in recent decades. Aristolochic acid I (AAI), an important ingredient, has been associated with tumorigenesis. Recently, some studies indicated that AAI could induce liver injury in mice of different age, but comprehensive mechanisms of AAI-induced differences in liver injury in various age groups have not yet been elucidated. This study aims to evaluate the causal relationship between AAI-induced liver injury and age based on neonatal mice and adult mice. A survival experiment indicated that all neonatal mice survived. Moreover, the adult mice in the high-dose AAI group all died, whereas half of the adult mice in the low-dose AAI group died. In observation experiments, AAI induced more severe liver injury in neonatal mice than adult mice under long-term than short-term exposure. Furthermore, integrated metabolomics and transcriptomics indicated that AAI disturbing steroid hormone biosynthesis, arachidonic acid metabolism, the drug metabolism-cytochrome P450 pathway and glycerophospholipid metabolism induced neonatal mice liver injury. The important role of age in AAI-induced liver injury was illustrated in our study. This study also lays a solid foundation for scientific supervision of AA safety.

## Introduction

Aristolochic acid (AA) is derived from certain plants, such as the genera Aristolochia and Asarum, which have been widely used as herbal remedies in traditional Chinese medicine (Yu et al., 2016; Zhang et al., 2021). However, AA has been discovered to be one of the most potent nephrotoxic human carcinogen, and it has been listed as a category-I carcinogen by the World Health Organization and the International Agency for Research on Cancer (Nault and Letouzé, 2019). Some studies have demonstrated that AA are metabolized into aristolochic acid lactam nitrogen ions with a strong electrophilic ability *in vivo*, allowing them to electrophilically combine with amino groups outside the DNA base ring to generate corresponding deoxyadenosine and deoxyguanosine adducts, to induce A-T transversion mutation of the gene product and form biomarkers related to the carcinogenic and mutagenic activity of aristolochic acids (Dračínská et al., 2016; Arlt et al., 2017; Stiborová et al., 2017; Liu et al., 2019). Recently, the correlation between AA and liver cancer has attracted widespread attention. One study indicated that AA can cause hepatocellular carcinoma (HCC) due to its genetic toxicity (Poon et al., 2013). An epidemiological study showed that there is a dose–response relationship between AA exposure and the development of HCC in patients with chronic HBV or HCV infection (Chen et al., 2018; Chi-Jen Chen et al., 2019). In a recent study, an AA-induced mutational signature (A-T transversion mutation) was found in 78% of 98 liver cancer patients in Taiwan (Ng et al., 2017).

In fact, AA can be divided into aristolochic acids I, II, III and IV, of which aristolochic acid I (AAI, C17H11NO7) is the main toxic component (Wang et al., 2017). However, in these early studies, AAI were demonstrated to inhibit carcinogenesis in carcinogen-induced animal models (Filitis and Massagetov, 1961; Kupchan and Doskotch, 1962; Jackson et al., 1964; Kupchan and Wormser, 1965). Herb containing AAI have been used for centuries to treat various human diseases (Dawson, 1927; Heinrich et al., 2009; Scarborough, 2011; Grollman and Marcus, 2016). In recent times, some scholars pointed out that AAI can induce the development of HCC. One study verified that canines had precancerous lesions in the liver after 10 days of oral administration of AAI (Jin et al., 2016). Another study indicated that exposure to short-term AAI *in vivo* and *in vitro* only showed a potential liver cancer-causing effect (Rossiello et al., 1993; Zhou et al., 2019). A recent paper demonstrated that AAI promoted clonal expansion but did not induce hepatocellular carcinoma in the liver of adult rats (Liu et al., 2021). Notably, liver tumors could be observed in neonatal mice after 9 months of AAI intake (Lu et al., 2020). Those studies have shown that AAI may be a risk factor for liver injury. However, current experimental or clinical studies on liver injury induced by AAI took no account of age, administration times, and treatment course (Sun et al., 2015; Chin-Nu Chen et al., 2019).

In this study, we considered the age stage at which AAI was used. Adult mice and neonatal mice mimicked adult and children, respectively. The treatment course is another important factor. Five days after the administration of AAI, short-term and long-term observation experiments were conducted to assess whether AAI could induce liver injury. Moreover, given that transcriptomics and metabolomics are ‘omics’ approaches widely employed to improve comprehension of the chemical mechanism of toxicity (Gupta et al., 2018), we combined metabolomics and genomics to explore the underlying mechanism of AAI-induced liver injury.

## Materials and Methods

### Chemical and Animal Treatment

Aristolochic acid I (CAS No. 313-67-7, ≥98.9%, MedChemexpress CO., Ltd, Shanghai, China) was dissolved in 4% dimethyl sulfoxide and 4% Tween-80 in 0.9% NaCl. The AAI exposure dose for neonatal rats were divided into low dose (2.5 mg/kg) and high dose (5 mg/kg). The low and high administration doses of adult mice were 2.2 mg/kg and 4.4 mg/kg, respectively.

**FIGURE 1:**
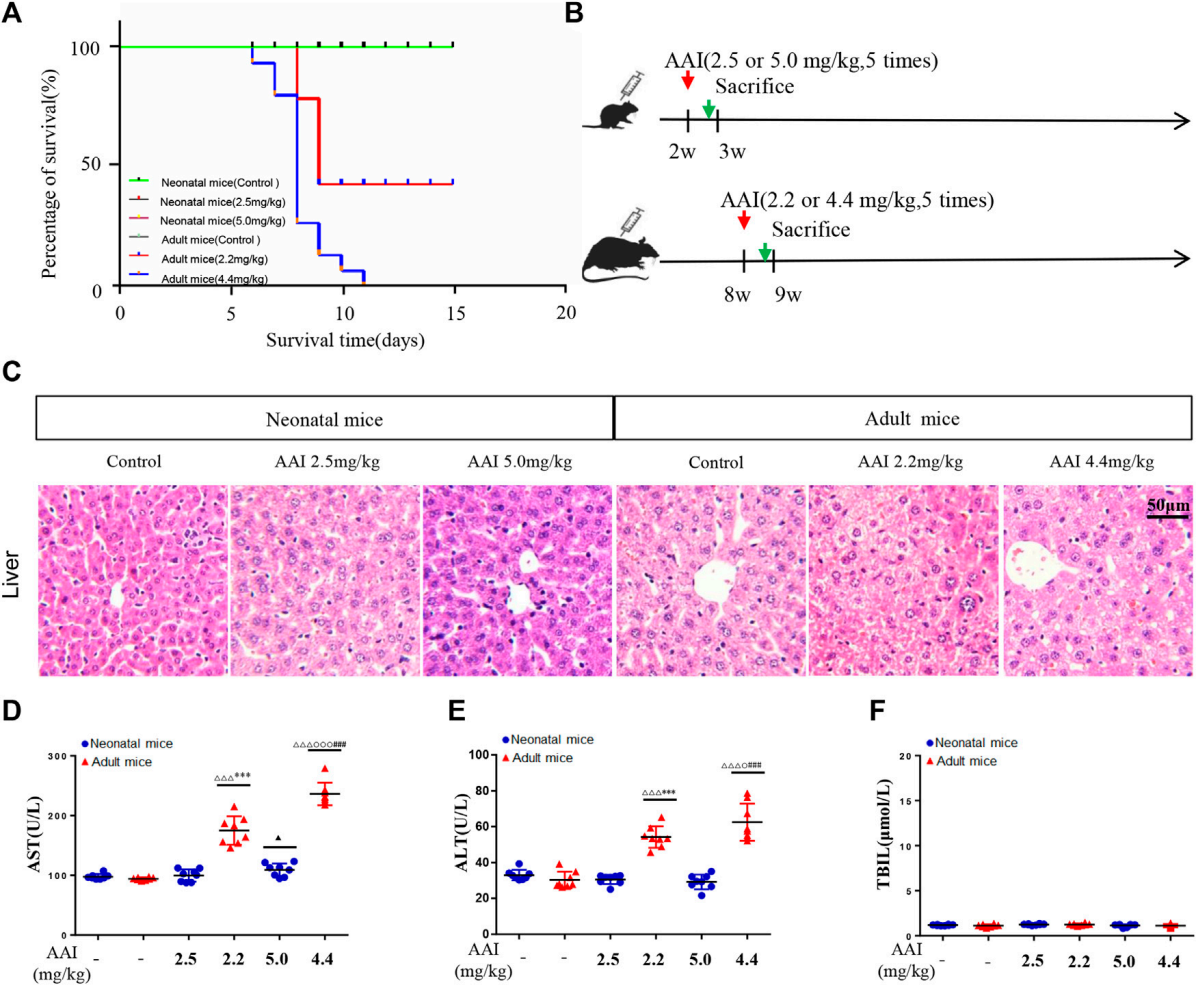
AAI induced indistinct liver injury in neonatal mice compared to adult mice based on a short-term observation study. **(A)** Survival curve of the animals subjected to AAI. **(B)** Schematic diagram of the study design. AAI was administered by intraperitoneal injection (once a day from Monday to Friday for a total of 5 days). **(C)** Representative microscopy images of hematoxylin and eosin-stained livers (scale bars, 50 μm). Clinical biochemical parameters between the control and AAI-treated groups: **(D)** aspartate aminotransferase; **(E)** alanine aminotransferase; **(F)** total bilirubin; Data are shown as the mean ± SD. Asterisks above a group between groups indicate a significant differences:^▲▲▲^ *p* < 0.001, ^▲▲^ *p* < 0.01, ^▲^ *p* < 0.05 • *vs. •*neonatal mice control group; ^△△△^ *p* < 0.001, ^△△^ *p* < 0.01, ^△^ *p* < 0.05 • *vs. •*adult mice control group;•••*p* < 0.001,••*p* < 0.01, •*p* < 0.05 • *vs. •*neonatal mice low-dose group;^○○○^ *p* < 0.001,^○○^ *p* < 0.01,^○^ *p* < 0.05 • *vs. •*adult mice low-dose group; ^***^ *p* < 0.001, ^**^ *p* < 0.01, ^*^ *p* < 0.05 neonatal • *vs. •*adult mice low-dose group;^###^ *p* < 0.001,^##^ *p* < 0.01,^#^ *p* < 0.05 neonatal • *vs.•*adult mice high-dose group.

**FIGURE 2:**
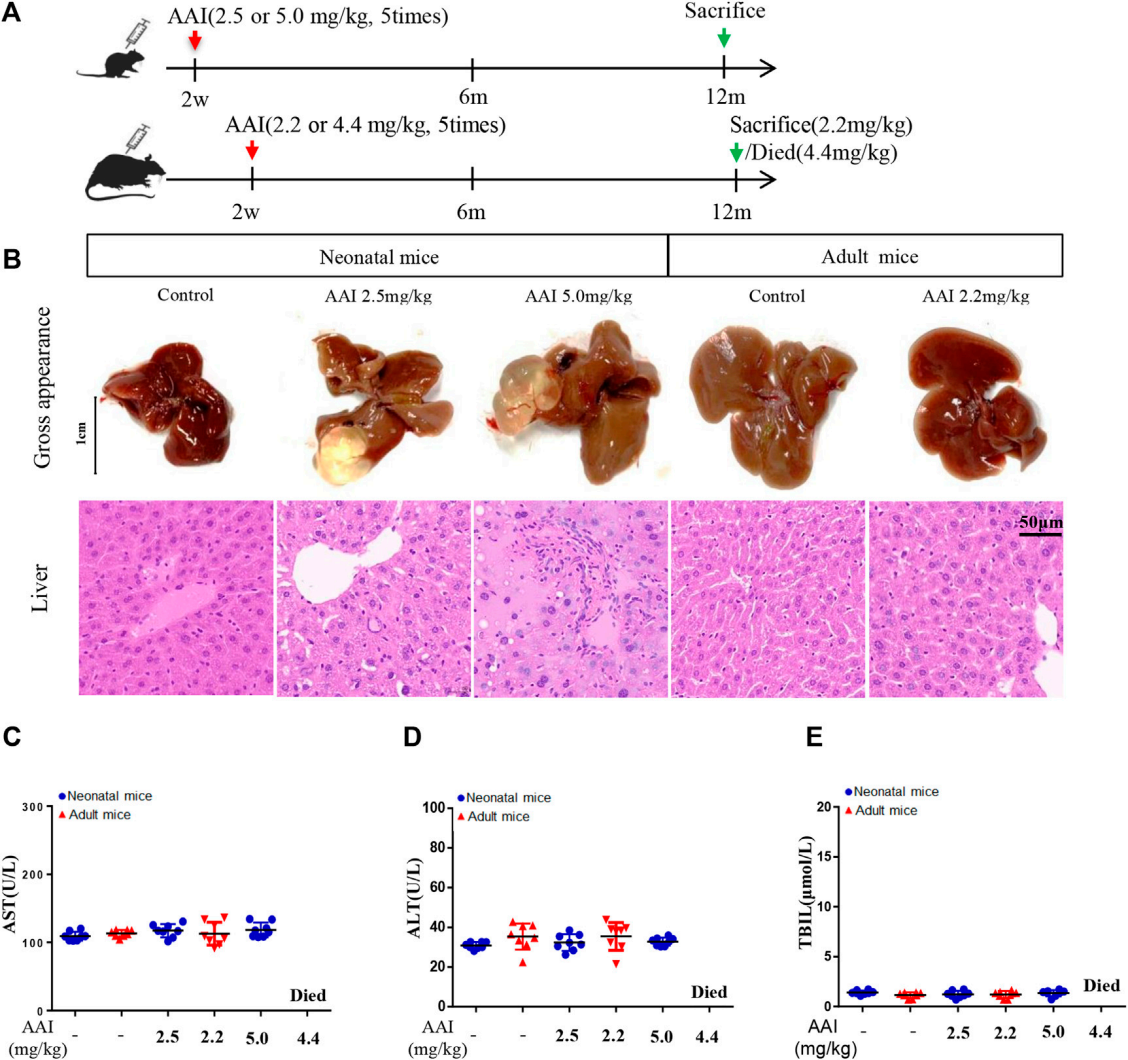
AAI induced serious liver injury in neonatal mice compared to adult mice based on long-term observation. **(A)** Schematic diagram of the study design. Fifty-two weeks of observation after AAI was administered by intraperitoneal injection (once a day from Monday to Friday for a total of 5 days). **(B)** Representative images of AAI-induced liver. The panel includes gross appearance (scale bars, 1 cm), and hematoxylin and eosin staining (scale bars, 50 μm). Clinical biochemical parameters: **(C)** aspartate aminotransferase; **(D)** alanine aminotransferase; and **(E)** total bilirubin. Data are shown as the mean ± SD. Asterisks above a group between groups indicate a significant differences: ^▲▲▲^ *p* < 0.001, ^▲▲^ *p* < 0.01, ^▲^ *p* < 0.05 • *vs. •*neonatal mice control group; ^**△△△**^ *p* < 0.001, ^△△^ *p* < 0.01, ^△^ *p* < 0.05 • *vs. •*adult mice control group;•••*p* < 0.001,••*p* < 0.01, •*p* < 0.05 • *vs. •*neonatal mice low-dose group;^○○○^ *p* < 0.001,^○○^ *p* < 0.01,^○^ *p* < 0.05 • *vs. •*adult mice low-dose group; ^***^ *p* < 0.001, ^**^ *p* < 0.01, ^*^ *p* < 0.05 neonatal • *vs. •*adult mice low-dose group;^###^ *p* < 0.001,^##^ *p* < 0.01,^#^ *p* < 0.05 neonatal • *vs. •*adult mice high-dose group.

All animal protocols were reviewed and approved by the animal ethics committee of the Fifth Medical Center of Chinese PLA General Hospital (IACUC No. 2021-0010). During the observation weeks, all mice were permitted water and food freely and held under a 12 h dark/light cycle at 22 ± 2°C. First, a survival study was designed to estimate toxicity in neonatal mice and adult mice. Eight-week-old male C57BL/6 mice (adult mice) and 2 week-old male C57BL/6 mice (neonatal mice) were purchased from SPF Biotechnology Co., Ltd. (Beijing, China). C57/6 neonatal mice and adult mice were randomly divided into three groups: the control group (*n* = 10), low-dose group (*n* = 10) and high-dose group (*n* = 10), all of which were fasted for 12 h before the experiment started. From Monday to Friday, the control, low-dose and high-dose groups were intraperitoneally injected with 0, 2.5 and 5.0 mg/kg/day AAI in the neonatal mice group, and with 0, 2.2 and 4.4 mg/kg/day AAI in the adult mice group. The survival status, number of sacrifices and time of sacrifice were all recorded. In the second study, 24 adult/neonatal mice were randomly divided according to the same standard as the previous three groups, with eight in each group, and the administration followed the same survival study protocol. Compared to the survival study, the exposure was shortened to 8 h after drug withdrawal (Figure 1B). In the third study, 24 neonatal mice were randomly divided according to the same standard as the previous three groups, with eight in each group; according to the survival study, 40 adult mice were randomly divided into three groups: control group (*n* = 8), low-dose group (n = 16) and high-dose group (n = 16). The administration followed the second study protocol, and the exposure was prolonged to 52 weeks after drug withdrawal (Figure 2A).

### Sample Collection

At the end of the study, blood samples were collected from the orbit of all treatment groups. The blood samples were centrifuged at 5,000 rpm for 20 min at 4°C to obtain serum samples, which were stored at −80°C before analysis. After blood collection, all mice were sacrificed, the tissue morphology of various organs was observed, and liver tissues were collected. Part of the liver in horizontal sections was immersed in 10% formalin, and the remaining liver was stored in liquid nitrogen.

### H and E Staining and Microscopic Examination

Mice liver samples were fixed in 10% formalin overnight and embedded in paraffin. The samples were sliced with a paraffin microtome with a thickness of approximately 5 μm, dyed with hematoxylin dye solution for 5 min, dehydrated and covered on slides with neutral gum, and observed through a microscope.

### Blood Biochemical Analysis

Serum samples of the treated and control groups were used for the analysis of blood aspartate aminotransferase (AST), alanine aminotransferase (ALT) and total bilirubin (TBIL). The samples were analyzed by an AU480 automatic biochemical analyzer produced by Japan Olympus Co, Ltd.

### Metabolomics Analysis Based on 52 weeks Observation

Each serum sample (100 µL) was added to 300 µL of frozen methanol (pre-cooling at −20°C for 20 min). After vortexing for 30 s, the samples were placed in a refrigerator at 4°C for more than 20 min to precipitate proteins and other solids. Then, the solution was centrifuged at 14,000 rpm for 10 min at 4°C, and the supernatant was transferred to a sample bottle.

UHPLC/MS-QTOF conditions: Ten microliters of sample was injected and analyzed with an Infinity 1290 UHPLC system (Agilent Technologies, SA, United States) and an Acquity UPLC HSS T3 C18 column (1.8, 2.1 × 100 mm, Agilent Technologies, SA, United States). The column temperature was set at 35°C. Sample separation was operated from 5 to 100% solvent B (acetonitrile) in solvent A (0.1% formic acid (v/v) in water) for 30 min at a flow rate of 300 nL/min. The mass data were obtained on an Agilent 6550A Q-TOF mass spectrometer (Agilent Technologies, SA, United States) under full scan mode (80–1,200 m/z) in both positive and negative ion modes using an Agilent Jet Stream ESI source. The electrospray ionization (ESI) source were operated in both positive and negative ion modes with the following settings: ion spray voltage was 4000 V in positive ion mode and 3000 V in negative mode, ion source gas 1 = 45 psi. Other source parameters were as follows: the temperature of the drying gas was set at 225°C; the flow rate was 11 L/min; and the voltage of the fragmentor, skimmer one and octupole RF peak were 230, 0 and 750 V, respectively. To ensure the stability and reproducibility of the liquid chromatography–mass spectrometry system, 10 μl of each sample was taken to obtain a quality control sample, which was then analyzed together with other samples. Repetition of quality control samples were performed four times in the system.

All of the LC-MS data were processed and exported through mzMine2, filtered and normalized through MetaboAlalyst 5.0, and *p* < 0.05 and fold change (FC) > 1.5 were set as the statistical significance thresholds. SIMCA14.1 software (Umetrics, Umea, Sweden) was used for multivariate analysis, including principal component analysis (PCA) and orthogonal partial least squares discriminant analysis (OPLS-DA). Variable importance in the projection value (VIP>1) and correlation coefficient value (|Pcorr| > 0.5) in the OPLS-DA analysis of metabolites were considered statistically significant. We choose the HMDB database (https://hmdb.ca/) to identify the compounds that exhibited significant changes as candidate biomarkers, and carried out pathway analysis of the potential biomarkers by MetaboAnalyst 5.0 (http://www.metaboanalyst.ca/) based on the Kyoto Encyclopedia of Genes and Genomes (KEGG) Pathway Library for mice (http://www.genome.jp/kegg/). The differences were considered significant when *p* < 0.05.

### Transcriptomics Analysis Based on 52 weeks Observation

First total RNA was extracted through the TRIzol method. Then agarose gel electrophoresis was conducted for RNA quality inspection, and finally the construction of a transcriptome library was conducted. AMPure XP beads were used to screen 200 bp cDNA sequences, PCR amplification was performed, AMPure XP beads were used again to purify the PCR products, and the library was finally obtained.

### Integrative Analysis of Multiomics Data Based on 52 weeks Observation

To conduct a comprehensive analysis of the transcriptome and metabolome, the differentially expressed genes (DEGs) and metabolites (DEMs) were mapped to the KEGG pathway database to obtain common pathway information. Cytoscape (Version 3.4.0) was utilized to visualize the relationship between the metabolome and transcriptome.

### Statistical Analysis

Experimental data were expressed as the mean ± standard deviation (mean ± SD). GraphPad Prism version 7.01 (GraphPad Software, Inc. Version 7.01) was used for data analysis. One-way analysis of variance (one-way ANOVA) was used for comparisons among groups. Two-sided *p* < 0.05, *p* < 0.01 or *p* < 0.001 indicated a significant difference.

## Results

### AAI Induces Indistinct Liver Injury in Neonatal Mice Compared to Adult Mice Based on a Short-Term Observation Study

The results of the survival study (Figure 1A) showed that adult mice that took the low-dose of AAI were all died on the third day after AAI withdrawal, and the final mortality rate was 53.3%. When adult mice were administered a high dose of AAI, they were died beginning on the first day after AAI administration for five consecutive days, and the mortality rate was 100% on the 11th day due to acute tubular necrosis. During this experiment, most adult mice expressed slow movements, listlessness and poor appetite symptoms before they were died; in contrast, all neonatal mice survived. This result suggested that when AAI was administrated at equivalent dose to neonatal and adult mice, the degree of damage of AAI to adult mice was significantly higher than that to neonatal mice. Figure 1C showed that no abnormality was observed in the livers of neonatal mice, but AAI exposure to adult mice induced mild inflammation. In addition, ALT, AST and TBIL were tested to further demonstrate the degree of liver injury, Figures 1D–F illustrated that ALT and AST were significantly increased in the AAI-treated adult group. Blood chemistry showed that AAI was prone to cause acute liver damage in adult mice in a short-term exposure, but had no obvious toxic effect on the corresponding organs of neonatal mice treated with equivalent dose. These results showed that AAI could result in slight liver injury to adult mice in the short term. Moreover, no visible injury was found in the stomach of these adult and neonatal mice, although acute renal tubular necrosis was observed both in low and high dosage of AAI-exposed adult mice (Supplementary Figure S1).

### AAI Induces Serious Liver Injury in Neonatal Mice Compared to Adult Mice Based on Long-Term Observation

In this study, we also conducted an observational experiment for 52 weeks. During the observation, all of the neonatal mice survived, whereas the AAI high-dose group of the adult mice were all died, and eight adult mice (8/16) were sacrificed in the AAI low-dose group, consistent with the survival experiment. In particular, hepatic cysts were observed in AAI-treated neonatal mice, three neonatal mice had hepatic cysts in the liver in the low-dose group, and four neonatal mice had hepatic cysts in the liver in the high-dose group (Figure 2B). However, there were no abnormalities in any of the adult mice groups (Figure 2B). The histopathology showed that (Figure 2B) the low-dose group and the high-dose group of neonatal mice exhibited hydropic degeneration or spotty necrosis. Moreover, there was only a small amount of pyknosis and nucleolysis of liver nuclei in the low-dose group of adult mice. Therefore, the degree of injuries of the neonatal mice with equivalent dose was more obvious than that of the adult mice. These results showed that AAI could result in liver injury in neonatal mice after a long-term exposure. Figures 2C–E showed that long-term exposed to AAI in neonatal and adult mice had minimal impact on the activity of AST and ALT or the concentration of TBIL. According to this finding, some studies considered that the unchanged blood chemistry indices of the liver may be related to the self-healing function of mice over a long time period (Nault and Letouzé, 2019). These results indicated that AAI could induce serious liver injury in neonatal mice compared to adult mice based on long-term observations. Furthermore, one study also demonstrated that if neonatal mice were exposed to 2.5 or 5 mg/kg AAI 3 times, 1 year later, they could demonstrate liver injury (Lu et al., 2020). Besides, acute tubular necrosis is the main cause of death in AAI-treated adult mice group, while no change was found in the kidney of AAI-treated neonatal mice group. Meanwhile no visible injury was found in the stomach of these adult and neonatal mice (Supplemenatary Figure S2).

### Metabolomics Analysis Based on Long-Term Observation

**FIGURE 3:**
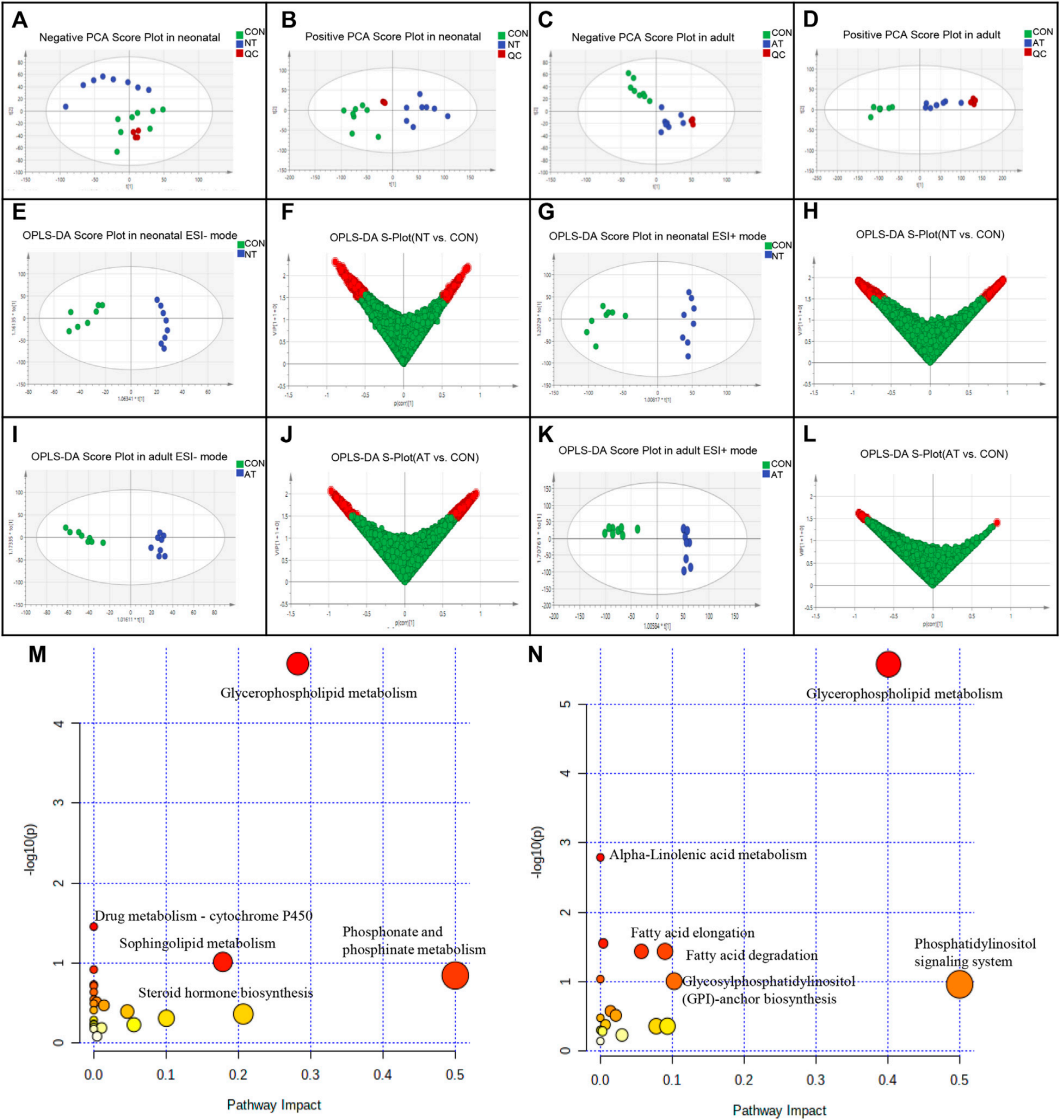
Metabolomic analysis of AAI-treated neonatal (NT) and adult mice (AT). Principal component analysis (PCA) scores for AAI-treated mice with the first two principal components: **(A)** negative ion mode of NT; **(B)** positive ion mode of NT; **(C)** negative ion mode of AT; **(D)** positive ion mode of AT. Discrimination of AAI-treated mice and control mice (CON) according to orthogonal projection to latent structures discriminate analysis (OPLS-DA) model in the ESI^−/+^ mode. The points in red indicate the identified biomarkers. **(E)** OPLS-DA score plots of NT and CON in the ESI- mode; **(F)** Score plot of the OPLS-DA model for the CON and NT; **(G)** OPLS-DA score plots of NT and CON in the ESI + mode; **(H)** Score plot of the OPLS-DA model for the CON and NT; **(I)** OPLS-DA score plots of AT and CON in the ESI- mode; **(J)** Score plot of the OPLS-DA model for the CON and AT; **(K)** OPLS-DA score plots of AT and CON in the ESI + mode; **(L)** Score plot of the OPLS-DA model for the CON and AT. Metabolome view of the pathway analysis generated using MetaboAnalyst 5.0 based on differential metabolites between AAI-treated mice and CON. The size and color of each circle were based on the pathway impact value and *p* value, respectively. **(M)** Summary of ingenuity pathway analysis in the NT group. **(N)** Summary of ingenuity pathway analysis in the AT group.

Serum metabolic profiling was assessed through a multivariate analysis among neonatal and adult mice in the control group and AAI low-dose group. The plots of PCA scores showed that untreated and AAI-treated neonatal and adult mice were completely distinguishable from one another (Figures 3A–D). Hence, as illustrated in the OPLS-DA plots (Figures 3E–L), metabolite profiles of the AAI-treated two groups were distributed in significantly separated clusters, in which R2Y and Q2Y were both greater than 0.5. A total of 38 differential endogenous metabolites were identified in the AAI-treated neonatal groups and 30 were identified in the AAI-treated adult group, in accordance with the following criteria. OPLS-DA VIP (variable importance in the projection) > 1.5, FC (fold change) > 1.5 and FDR (false discovery rate) < 0.05, and the specific information (KEGG ID, RT, measured m/z, formula, P, FDR, Log2 (FC), VIP values) for these metabolites was summarized (Supplementary Tables S1, 2).

We identified 38 differential metabolites (25 upregulated/13 downregulated) (Supplementary Table S1) and 30 differential metabolites (10 unregulated/20 downregulated) (Supplementary Table S2) were significantly affected by AAI in neonatal mice and adult mice, respectively. Most of the differential metabolites were classified into lipids and glycerolipids, such as PC (phosphatidylcholine), PE (phosphatidylethanolamine), PS (phosphatidylserine), PGP (phosphatidylglycerophosphate), and PG (phosphatidylglycerol), which showed obvious decreases in both groups after AAI exposure. However, most metabolites were different between AAI-treated neonatal mice and adult mice, indicating that these specially changed metabolites were associated with AAI-induced liver injury.

We utilized MetaboAnalyst 5.0 to analyze metabolic pathways and studied the effect of AAI on related pathways perturbed in neonatal and adult mice (Supplementary Tables S3,4). These results revealed that the significantly difference metabolites were mainly associated with glycerophospholipid metabolism and drug metabolism - cytochrome P450, in accordance with specific criteria (raw *p* < 0.05) (Figure 3M) in the AAI-treated neonatal mice group. The AAI-treated adult mice group was mainly related to metabolic pathways connected with glycerophospholipid, glycosylphosphatidylinositol (GPI)-anchor biosynthesis, linolenic acid, fatty acid elongation and degradation, in accordance with specific criteria (raw *p* < 0.05) (Figure 3N).

### Transcriptomics Analysis Based on Long-Term Observation

**FIGURE 4:**
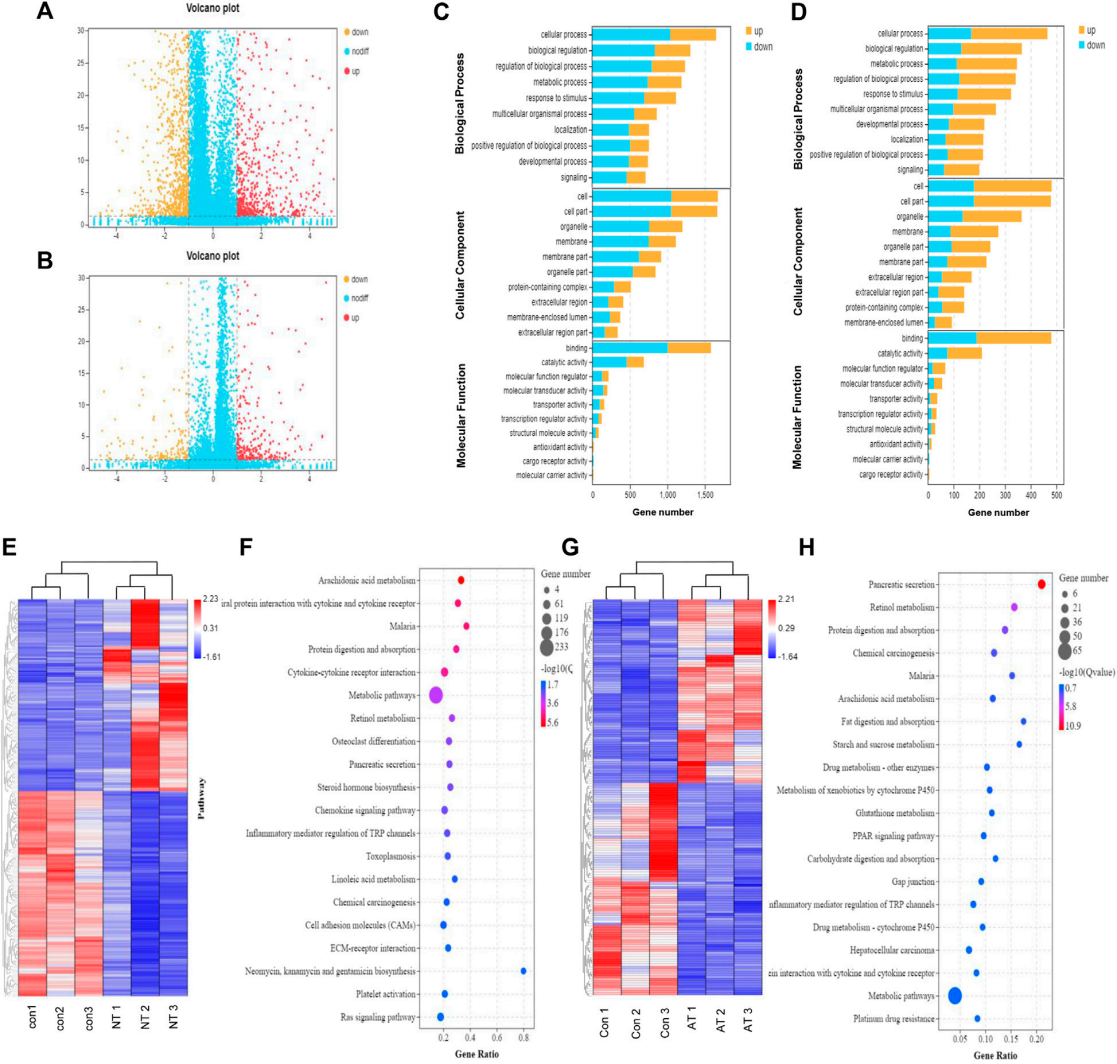
Transcriptomic analysis of AAI-treated neonatal (NT) and adult mice (AT). Volcano graph showed the differentially expressed genes (DEGs) in the NT group **(A)** and AT group **(B)**. The red dots represent significantly upregulated genes, and the orange dots represent significantly downregulated genes. Enriched GO terms for DEGs in the NT group **(C)** and AT group **(D)**. The ontology covered three domains: biological process, cellular component, and molecular function; the *Y*-axis shows gene functions, and the *X*-axis corresponds to gene numbers. Heatmap displayed the DEGs in the replicate NT group **(E)** and AT group **(G)** compared to the CON group. The columns showed the genes that were significantly upregulated (red) or downregulated (blue) in the different groups. Functional pathway enrichment of DEGs in the NT group **(F)** and AT group **(H)** using the KEGG database. (The most enriched top 20 pathway terms).

To confirm the transcriptomic changes underlying the effect of low-dose AAI on neonatal and adult mice, RNA-seq data were generated from treatment and control samples. The volcano plot clearly reflected the obviously expressed genes with red and blue color in the two groups. As a result, 2161 genes were identified to have significantly changed expression after AAI treatment in the neonatal group, and the expression was upregulated in 850 genes and downregulated in 1311 genes (*p* < 0.05; FC > 2.0) (Figure 4A, Supplementary Table S5). Furthermore, 643 genes were identified to have significantly changed expression after AAI treatment in the adult group, and expression was upregulated for 411 genes and downregulated for 232 genes (*p* < 0.05; FC > 2) (Figure 4B, Supplementary Table S6). A heatmap illustrated differential expression patterns of transcripts, highlighting clusters of transcripts with significantly differential expression patterns between the treatment and control groups (Figures 4E–G). These results indicated that AAI has a significant effect on the transcription of neonatal mice and adult mice. Based on gene function annotation, some DEGs involved in inflammatory metabolism were observed, such as PLA2 (phospholipase A2), ALOX5 (arachidonate 5-lipoxygenase), PTGDS (prostaglandin D2 synthase), and PTGS1 (prostaglandin-endoperoxide synthase 1) were significantly changed in the AAI-treated neonatal mice group. In addition, detoxification-related genes, such as GPX6 (glutathione peroxidase 6), GGCT (gamma-glutamylcyclotransferase), MGST3 (microsomal glutathione S-transferase 3), GSTA1 (glutathione-s-transferase alpha-1), and GSTT3 (glutathione-s-transferase theta-3) were significantly changed after AAI exposure in the adult mice group.

Based on the GO and KEGG databases, transcriptome changes were analyzed to further characterize DEGs at an overall level through pathway-enrichment analyses. GO functional annotation of the DEGs revealed that the influence of AAI exposure was closely connected to biological processes such as cell morphology and exercise, and the effect on neonatal mice was greater than that on adult mice (Figures 4C,D). Additionally, KEGG pathway-enrichment analysis demonstrated that AAI treatment in the neonatal group influenced genes that were connected significantly with pathways related to arachidonic acid metabolism, protein digestion and absorption, steroid hormone biosynthesis, chemokine signaling pathway, and inflammatory mediator regulation of TRP channels (Figure 4F). The transcripts regulated in AAI-treated adult mice could be mapped to signaling pathways, such as protein digestion and absorption, arachidonic acid metabolism, fat digestion and absorption, metabolism of xenobiotics by cytochrome P450, and glutathione metabolism (Figure 4H). All of the data demonstrated that genetic expression was altered significantly in treated neonatal and adult mice.

### Integrated Analysis of Metabolomic and Transcriptomic Data

**FIGURE 5:**
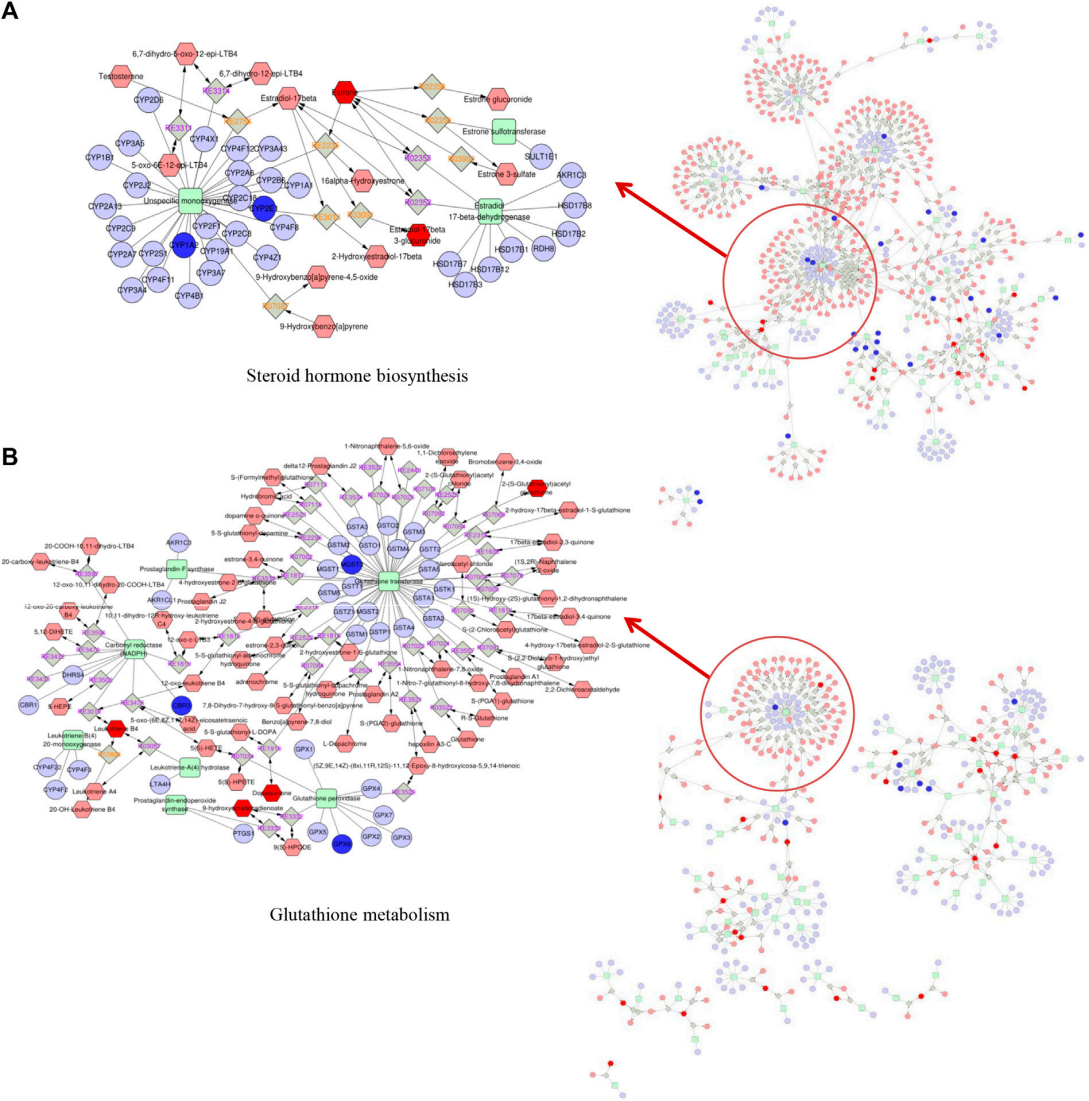
Metabolic pathways were visualized by means of Cytoscape software. Integrated analysis of the mechanism underlying the effects of AAI treatment on mice based on metabolomic and transcriptomic data by means of Cytoscape software. **(A)** Network built in AAI-treated neonatal mice. **(B)** Network built in AAI-treated adult mice. The metabolic biomarkers are represented by red hexagons and genic biomarkers are represented by blue circles in our study.

To further illustrate the mechanism, we evaluated the underlying relationship between genes and metabolites by performing an integrated pathway analysis. We performed a gene–metabolism network through Cytoscape to elaborate the relationship between gene regulation and metabolic changes (Figure 5). Integrative omics analysis clearly demonstrated that four KEGG pathways were shared between neonatal and adult mice, including the arachidonic acid metabolism pathway, drug metabolism-cytochrome P450 pathway, glycerolipid metabolism pathway, and linoleic acid metabolism pathway. In addition to shared integrated pathways, steroid hormone biosynthesis was notably observed in the AAI-treated neonatal group (Figure 5A) and glutathione metabolism was found in the AAI-treated adult mice group (Figure 5B). These integrated metabolomic and transcriptomic data demonstrated that significant metabolite pathways were indicative of AAI-induced liver injury.

## Discussion

AA has been commonly reported to treat various diseases such as snakebites, diarrhoea and gynaecological conditions, for centuries (Michl et al., 2014). In recent decades, it has been confirmed that AA can induce renal toxicity and cause aristolochic acid nephropathy (AAN) (Schmeiser et al., 2014; Han et al., 2019; Anger et al., 2020). Since 2017, AA has also induced HCC in Chinese and Southeast Asian populations based on clinical data analysis (Ng et al., 2017). Some studies have shown that AAI, the most dominant component of AA, could induce liver injury in adult rodent animals (Nault and Letouzé, 2019; Li et al., 2020). Notably, a previous study indicated that AAI could induce liver cancer in neonatal mice through a rodent model of 2 week-old mice for safety evaluation (Lu et al., 2020). However, most experimental studies based on the relationship between AAs and HCC did not consider pediatric patients (Sun et al., 2015; Ng et al., 2017; Chi-Jen Chen et al., 2019). Herbal medicines containing AA are also commonly used to treat cough, fever and diarrhea in children (Han et al., 2019). Therefore, the study of an adult mice model alone cannot fully illuminate the problem of liver injury caused by AAI. Based on a prophase investigation, age was fully considered in this study, and we constructed neonatal and adult liver injury mice model to comprehensively evaluate the objectivity of liver injury induced by AAI.

The short-term effects of AAI exposed on neonatal and adult mice were investigated. The results showed that at equivalent doses, acute liver injury in adult mice in the short term after AAI administration was more obvious than that in neonatal mice. AST and ALT remarkably increased in adult mice after 5 days of administration, but not in neonatal mice. There was no acute liver injury in neonatal mice within a short time after administration, which might be related to the lower activity of metabolic enzymes in neonatal mice than in adult mice (Fu et al., 2000). Furthermore, we performed long-term (52 weeks) observations to assess the degree of AAI-induced liver injury. AAI resulted in liver cysts in the neonatal group, and all AAI-treated adult mice were died successively after high-dose administration for 5 days, at the same time 50% of mice were died in the low-dose group of adult mice during the observation period. Some literatures also indicated that AAI could induce adult mice sacrifice through acute renal tubular injury (Sato et al., 2004; Hu et al., 2017). Moreover, the surviving adult mice might progress to chronic kidney disease (CKD) after a recovery period due to maladaptive repair or other underlying mechanisms (Ogbadu et al., 2019). We measured AST, ALT and TBIL to evaluate the degree of liver injury, but there was no significant change in either neonatal or adult mice. Similarly, one study reflected that AST or ALT decreased to the level of the control group on the 14th day after oral-administration of 30 mg/kg AAI in male SD rats (Zhou et al., 2019). The findings suggested that liver cells were damaged in the early stage and may be repaired in the later stage.

**FIGURE 6:**
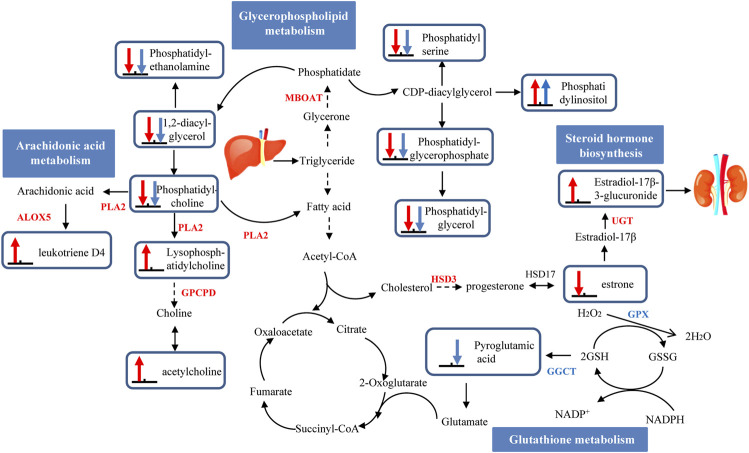
A schematic presentation of hepatotoxicity pathways identified by multiomics induced by AAI in neonatal and adult mice. The identified metabolites and genes involved in neonatal mice pathways are marked in red, and adult mice are marked in blue. Up arrows indicate upregulation, and down arrows indicate downregulation. Abbreviations: ALOX5: arachidonate 5-lipoxygenase; CEL: bile salt-stimulated lipase; GSH: reduced glutathione; GSSG: oxidized glutathione; GGCT: gammaglutamylcyclotransferase; GPX: glutathione peroxidase; LIPC: phosphatidate phosphatase; MBOAT: lysophospholipid acyltransferase; PLA2: phospholipase A2; PNLIP: pancreatic triacylglycerol lipase; PNPLA: patatin-like phospholipase domain-containing protein.

Advancement of high-throughput technologies has been adopted to more deeply and comprehensively explain the mechanisms of diseases. The integration of transcriptome and metabolome data may be particularly informative to supply novel insight into the potential mechanism of liver injuries caused by AAI (Cavill et al., 2016). Furthermore, we established a hypothetical network to elaborate upon the mechanism by which AAI-induced liver injury occurs in neonatal mice but not in adult mice (Figure 6).

With long-term exposure to AAI, no liver injury in adult mice was observed, which was tentatively attributed to the drug metabolism-cytochrome P450 pathway and glutathione metabolism pathway. The metabolic activity of the compound needs to be biotransformed by the phase I enzyme cytochrome P450 (CYP450), and then the phase II enzyme combines with GST (glutathione-s-transferase) to catalyze the detoxification reaction and excretion (Stiborová et al., 2014). A number of studies on the metabolic mechanism of AAI have elucidated that the CYP450 enzyme plays an important role in the process of detoxification (Stiborová et al., 2015). CYP1A2 is a critical hepatic member of the CYP450 family. The process by which AAI is demethylated by CYP1A1/2 to produce AAIa is considered to be the detoxification process of AAI (Reed et al., 2018). However, AAI-treated adult mice did not exhibit inhibited activity of liver microsomal CYP1A2. Additionally, glutathione has been shown to play a critical role in detoxification processes (Stiborová et al., 2017). AAI inhibited GSH conversion into pyroglutamic acid (by -2.0679-fold) by interfering with the expression of GPX (by -3.8496-fold), and reduced the oxidation of GSH by downregulation GGCT (by -1.6934-fold). In summary, the level of GSH was increased in this pathway, which played a crucial protective role against injury when adult mice were exposed to AAI.

During long-term exposure, liver injury generated in AAI-neonatal mice was due to the drug metabolism-cytochrome P450 pathway, glycerophospholipid metabolism, arachidonic acid metabolism and steroid hormone biosynthesis pathway. In the drug metabolism-cytochrome P450 pathway, the downregulation of CYP1A2 (by 1.0713-fold) among AAI-treated neonatal mice inhibited the detoxification of AAI, leading to its accumulation in the body and causing liver damage. Then, in the AAI-treated neonatal mice, AAI disturbed the phospholipid metabolic process and promoted arachidonic acid metabolism to release inflammatory factors. A previous study revealed that phospholipase A2 (PLA2) is responsible for catalyzing phosphatidylcholine transfer to hemolytic lecithin, accompanied by the release of arachidonic acid and fatty acids (Zhou et al., 2019). In the metabolic pathway, phosphatidylcholine was converted into LysoPC (22:4 (7Z, 10Z, 13Z, 16Z)/0:0) (by 1.9735-fold) and arachidonic acid to generate leukotriene D4 (LD4) (by 3.4274-fold). The increased LD4 might activate inflammation to promote liver damage in the AAI-treated neonatal group (Nanji et al., 1993). Interestingly, we also found that the steroid hormone biosynthesis pathway was more important in liver injury in AAI-treated neonatal mice. Some studies have described that the regulation of estrogen plays a protective role in elements of HCC (Villa, 2008). An epidemiological study indicated that men and menopausal women were more sensitive than women to induction of liver cancer, which was related to the lack of estrogen (Liu, 2014). In the steroid hormone biosynthesis pathway among in AAI-treated neonatal mice, The upregulated expression of the UGT enhanced estradiol metabolism and increased the excretion level of 17-beta-estradiol-3-glucuronide (by 2.0166-fold); AAI also inhibited the expression of the HSD3 gene to decrease the level of estrone (by -3.1339-fold), the transformation material of estradiol. Therefore, we inferred that estradiol may exhibit no protective effects on liver injury in AAI-treated mice by reducing its the formation. These results supported the role of the steroid hormone biosynthesis pathway as a potentially critical biological process that may be connected with the development of liver cysts among AAI-treated neonatal mice.

## Conclusion

Our studies showed that when exposed to equivalent doses of AAI, adult mice and neonatal mice could suffer different degrees of liver injury during different exposure times. Acute liver injury was generated in adult mice compared with neonatal mice based on short-term observation. We performed multiomics analysis to elucidate the mechanism. AAI-induced liver injury in neonatal mice may be mainly related to steroid hormone biosynthesis, whereas glutathione may protect adult mice from liver injury during the long-term withdrawal period in the glutathione metabolism pathway. In our study, age and medication time were comprehensively considered to evaluate the degree of liver injury, which is more consistent with clinical practice.

## Data Availability

The datasets presented in this study can be found in online repositories. The names of the repository/repositories and accession number(s) can be found below: https://www.ncbi.nlm.nih.gov/sra/PRJNA788199.
