# Supplementary figures and images for: Integration of Transcriptomic and Metabolomic Data to Compare the Hepatotoxicity of Neonatal and Adult Mice Exposed to Aristolochic Acid I
Source: Front Genet. 2022 Mar 25;13:840961. doi: 10.3389/fgene.2022.840961 (PMC8992794; doi:10.3389/fgene.2022.840961)

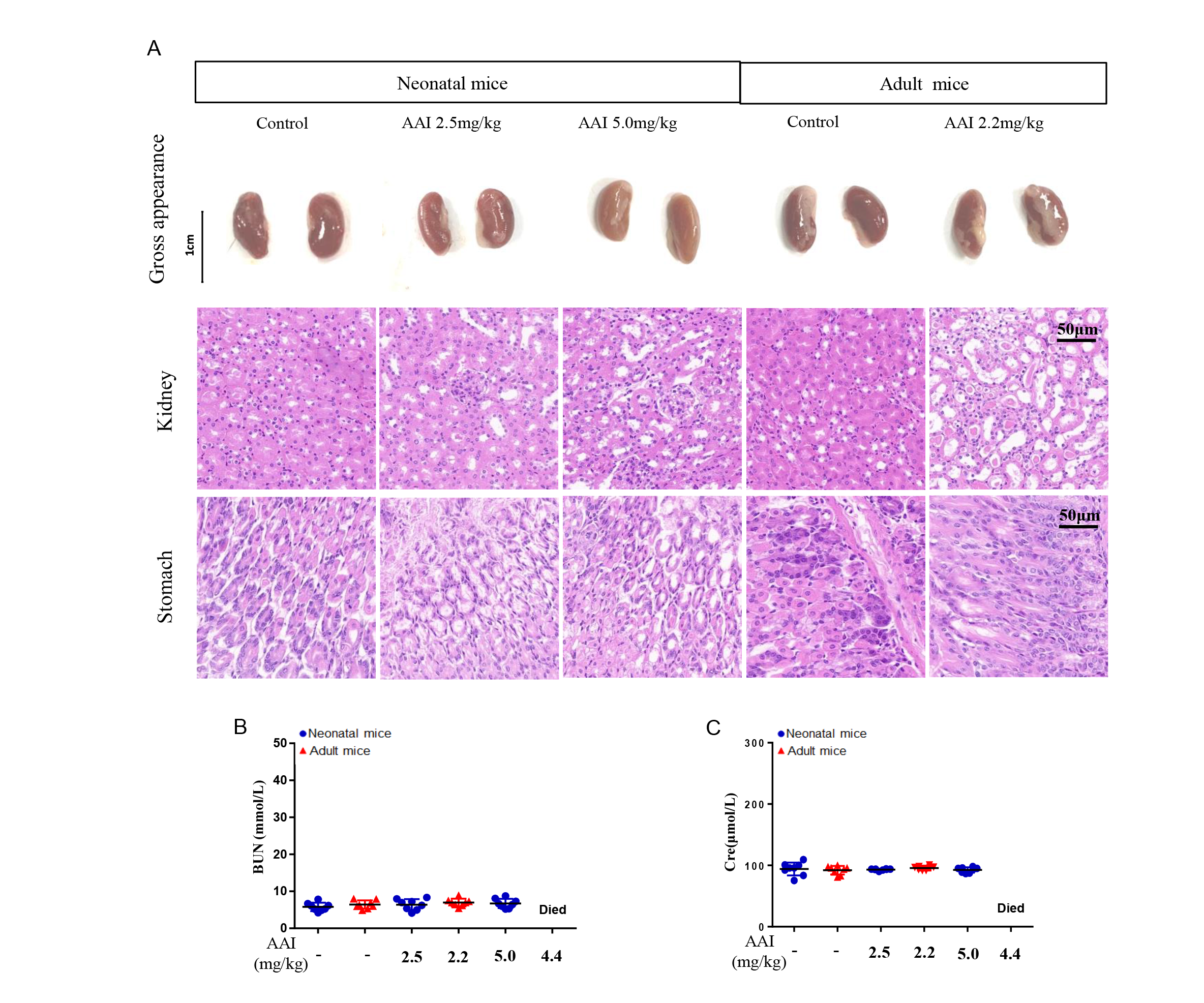

Supplement: Supplementary file 4 [file Image2.tif]

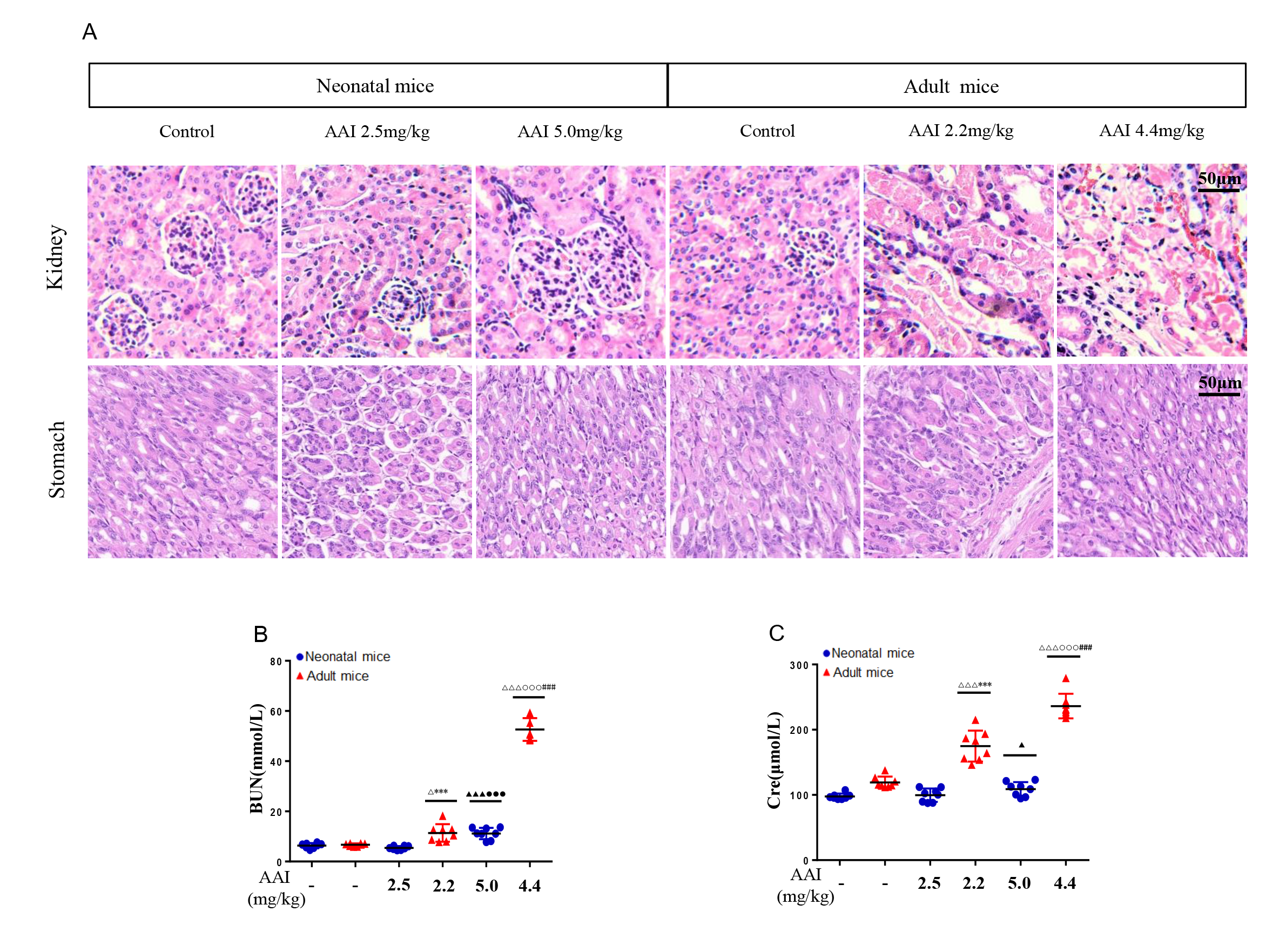

Supplement: Supplementary file 5 [file Image1.tif]
